# Glutathione S-transferasesP1 AA (105Ile) allele increases oral cancer risk, interacts strongly with c-Jun Kinase and weakly detoxifies areca-nut metabolites

**DOI:** 10.1038/s41598-020-63034-3

**Published:** 2020-04-07

**Authors:** Pallavi Yadav, Atanu Banerjee, Nabamita Boruah, Chongtham Sovachandra Singh, Puja Chatterjee, Souvik Mukherjee, Hughbert Dakhar, Henry B. Nongrum, Atanu Bhattacharjee, Anupam Chatterjee

**Affiliations:** 1 Molecular Genetics Laboratory, Department of Biotechnology & Bioinformatics, North-Eastern Hill University, Shillong, Meghalaya 793022 India; 2 Oral and Maxiofacial Surgery, MR Ambedkar Dental College and Hospital, Cooke Town, Bengalore, 560004 Karnataka India; 3 National Institute of Biomedical Genomics, Kalyani, 741251 West Bengal India; 4 Histopathology Division, Nazareth Hospital, Laitumkhrah, Shillong, 793003 India; 5 Otolaryngology and Head and Neck Surgery Department, Nazareth Hospital, Laitumkhrah, Shillong, 793003 India

**Keywords:** Cancer genetics, Computational biology and bioinformatics

## Abstract

The Glutathione S-transferases (GSTs) protects cellular DNA against oxidative damage. The role of GSTP1 polymorphism (A313G; Ile105Val) as a susceptibility factor in oral cancer was evaluated in a hospital-based case-control study in North-East India, because the habit of chewing raw areca-nut (RAN) with/without tobacco is common in this region. Genetic polymorphism was investigated by genotyping 445 cases and 444 controls. Individuals with the GSTP1 AA-genotype showed association with the oral cancer (OR = 3.1, 95% CI = 2.4–4.2, p = 0.0002). Even after adjusting for age, sex and habit the AA-genotype is found to be significantly associated with oral cancer (OR = 2.4, 95% CI = 1.7–3.2, p = 0.0001). A protein-protein docking analysis demonstrated that in the GG-genotype the binding geometry between c-Jun Kinase and GSTP1 was disrupted. It was validated by immunohistochemistry in human samples, showing lower c-Jun-phosphorylation and down-regulation of pro-apoptotic genes in normal oral epithelial cells with the AA-genotype. *In silico* docking revealed that AA-genotype weakly detoxifies the RAN/tobacco metabolites. In addition, experiments revealed a higher level of 8-Oxo-2′-deoxyguanosine induction in tumor samples with the AA-genotype. Thus, habit of using RAN/tobacco and GSTP1 AA-genotype together play a significant role in predisposition to oral cancer risk by showing higher DNA-lesions and lower c-Jun phosphorylation that may inhibit apoptosis.

## Introduction

The oral squamous cell carcinoma (OSCC) is the most common cancer in India, with highest occurrence in the north-eastern part of the country^1^. The traditional habit of chewing raw rather than dry areca-nut with lime appears to be an important causative factor in addition of tobacco^2,3^. In North-East India, particularly in Meghalaya, the betel-quid contains raw and unprocessed areca-nut (RAN), lime paste and small portion of betel-leaf but without tobacco. It has been noted that alkaloids, and polyphenols and tannic acid of RAN that are released in the saliva may contribute to carcinogenicity^4–6^. It has been demonstrated that areca-nut alkaloids cause depression of antioxidants including glutathione and glutathione-S-transferases (GST) that are known to neutralize reactive oxygen species^7^. Earlier studies indicate that RAN and lime together induce oral, esophagous and stomach cancers both in mouse and human and highlighted the occurrence of precocious anaphase (premature separation of sister-chromatids) and higher expression of p53 and Securin as a potential screening marker for identification of mitotic checkpoint defects during early days of RAN exposure^8,9^.

There are enough data to view lifestyle as well as genetic factors as important contributors for an individual’s susceptibility to cancer^10^. Glutathione redox and GST are supposed to play important roles in cellular detoxification^11^. GSTs protect cellular macromolecules from attack by reactive electrophiles, including environmental carcinogens and reactive oxygen species^11^. The human cytosolic GST family, comprising 16 genes belonging to 8 distinct classes, is well studied and considered to be relevant to various disease manifestations^12,13^. It has been demonstrated in Assam and other regions in North-East India that GSTM1 null genotype is associated with esophageal cancer in fermented areca-nut chewers whereas smoking and alcohol do not show any association either with this or GSTT1 genotypes^14^. Moreover, single nucleotide polymorphisms (SNPs) in both the coding and the regulatory region of these genes may alter their enzymatic activity and increase the risk of certain cancers^12,13,15,16^.

Polymorphism in GSTP1 due to A to G transition at nucleotide 313 (A313G) of the coding region (rs1695) leads to substitution of 105^th^ amino acid isoleucine (Ile) with valine (Val). The wild-type homogeneous AA genotype of GSTP1 shows the highest enzymatic activities^17^. However, such non-synonymous SNP either changes the activity or its affinity with the substrate^18,19^ and considered to be a risk factor for the breast^15^, head and neck^16^ and hepatocellular carcinoma^20^. Interestingly, in other studies, GSTP1 AA-genotype (Ile/Ile) has been reported to be associated with the risk of esophageal^21^ and oral cancers^22^ in smokers and tobacco chewers. The presence of GSTP1 AG/GG genotype shows protective effect against cervical cancer with a better survival advantage^23,24^.

Besides cellular detoxification, GSTP1 plays an important role in modulating activities of other enzymes through protein-protein interactions. For instance, it is an inhibitor of c-Jun N-terminal kinase (JNK) whose activation leads to c-Jun phosphorylation^25^. GSTP1-mediated JNK inhibition occurs in a dose-dependent manner with up to 80% inhibition of its c-Jun kinase activity has been reported in mouse fibroblast cell line^26^.

It is true that GST gene family has been studied extensively and decreased detoxification capacity of GSTP1 rs1695 (A313G) has also been demonstrated earlier^27^. GSTP1 has the potential for detoxification by conjugating various metabolites of RAN/tobacco with reduced GSH and therefore it is possible that GSTP1 genotypic variations (A313G) with consequential lower enzyme activities, may modify susceptibility to RAN/tobacco metabolites. A total of thirteen metabolites of RAN alkaloids^28^ and nine metabolites of tobacco^29^ have been identified in the urine of areca-nut-chewers and tobacco-users through a metabolomics approach. Hence, it is reasonable to conduct a systematic characterization of the variation in this gene and derive its functional significance. Thus, in order to unravel the role of GSTP1 as a susceptibility factor in oral oncogenesis, the present study investigates the interaction of the complex genotypes/phenotypes of GSTP1 with JNK and different xenobiotics. We have explored the association of GSTP1 (A313G) (rs1695) with the risk of oral cancer in the people chewing RAN with and without tobacco. Having established the correlation, we have evaluated the interaction between GSTP1 and JNK through protein-protein docking analysis and subsequently validating experimentally for the first time in human samples. In addition, the efficiency of detoxification of GSTP1 AA- and GG-genotypes on different RAN/tobacco metabolites has been assessed through *in silico* docking approach. Validation of these observations has been done experimentally by quantitation of 8-Oxo-2′-deoxyguanosine (8-OHdG), a known marker of oxidative stress-mediated damage to DNA^30^ in DNA digests from tumor tissues and blood lymphocytes.

## Results

### General characteristics of the included subjects

Details of the patients and tumor characteristics in the current study are summarized in Supplementary Table (S1). Cases (mean ± SD) are slightly older than controls (mean ± SD). During analysis, the variables like age, gender and habit are adjusted appropriately.

### GSTP1 AA-genotype is associated with the oral cancer risk

The rs1695 is found to be polymorphic in the oral cancer patients and in controls i.e., Minor Allele Frequency ≥ 0.05 (Table 1). The present SNP does not show any deviation from Hardy-Weinberg Equilibrium in the control group (Table 1). The present data demonstrate that GSTP1 AA-genotype is significantly associated with the oral cancer in cases compared to controls (Dominant model; OR = 3.1, 95% CI = 2.4–4.2, p-value = 0.0002) (Table 1). Even after adjusting for age, sex and habit the AA-genotype is found to be significantly associated with the risk of oral cancer in cases compared to controls (OR = 2.4, 95% CI = 1.7–3.2, p = 0.0001) (Table 1). To investigate the contribution of risk genotypes of rs1695 in oral cancer, we performed habit-matched regression analysis separately for two groups including individuals with two different habits “RAN Only” and “RAN + tobacco”. Even after adjusting for the probable confounders, the significant association of AA-genotype with oral cancer risk in cases is still evident (OR = 2.3, 95% CI = 1.4–3.7, p = 0.0001 for RAN only group; OR = 2.4, 95% CI = 1.6–3.7, p = 0.0001 for RAN + tobacco group) (Table 2).

**Table 1:** Comparison of genotype frequencies of rs1695 in GSTP1 gene between oral cancer patients and healthy controls.

SNP ID	Gene Name	Alleles	Healthy Control Group						Oral Cancer Group					Odds ratio (95%CI)^a^	
			Genotype Frequencies				Minor allele freq	p-value (HWE)	Genotype counts				Minor allele freq	Unadjusted p-value^b^	Adjusted p-value^c^
			n	AA	AG	GG			n	AA	AG	GG			
rs1695	GSTP1	A > G	444	213	195	36	0.3	>0.05	445	332	98	15	0.14	3.1 (2.4–4.2) 0.0002^d^	2.4 1.7–3.2) 0.0001^d^

^a^The odds ratio was calculated for the common genotype (AA) with reference to the variant genotype (AG + GG); Dominant model.

^b^Chi-square p-value estimated by comparing genotype frequencies between cases and controls.

^c^Odds ratio and p-value for multivariate logistic regression adjusted for age, sex and habit between cases and controls.

^d^p-value < 0.05 is considered to be significant.

**Table 2:** Multivariate Binary Logistic Regression Analysis with habit-matched to identify risk genotypes associated with oral cancer in rs1695 of GSTP1 gene.

Habit	Genotypes	No. of Controls	No. of Cases	Adjusted OR (95% CI)	p-value
RAN only	AA	69	139	2.4 (1.5–3.9)^a^	0.0001^1^
	AG + GG	93	59	Reference	
RAN + Tobacco	AA	126	195	2.4 (1.6–3.6)^1^	0.0001^1^
	AG + GG	84	54	Reference	

OR: odds ratio; 95% CI: 95% confidence interval; p-value <0.05 is considered to be significant.

^a^The Odds ratio and p-value were estimated after adjusting age and sex

### Mutant protein modelling, structure validation and its comparison with wild protein

**Figure 1:**
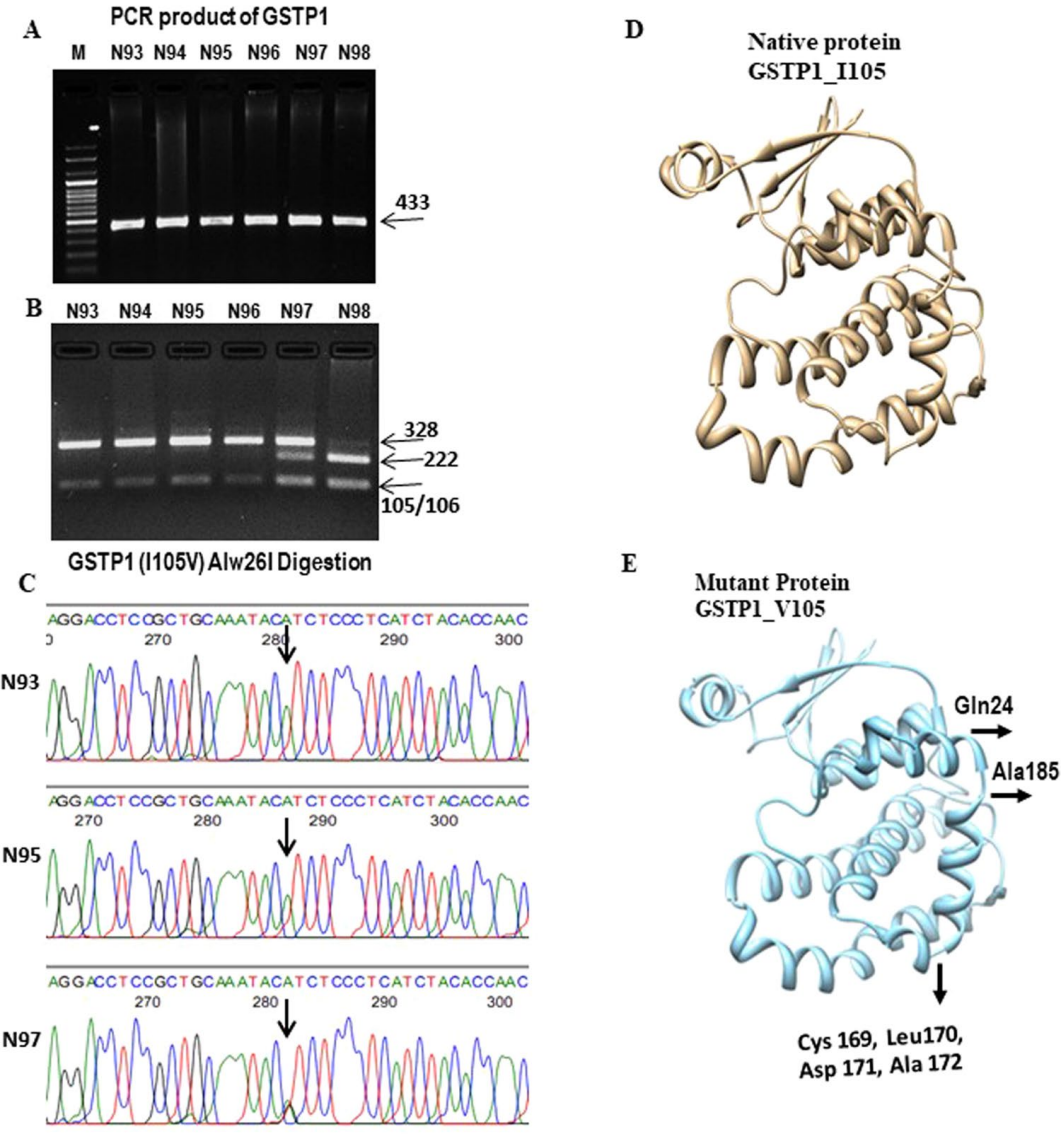
Analysis of SNP of *GSTP1* A313G and its 3D monomer structure. (**A**) A complete gel picture of the PCR-product of GSTP1 from six samples; M-marker. (**B**) A complete gel picture shows the result of PCR-RFLP analysis for GSTP1 SNP. The presence of *Alw26I* restriction site yielded 328 and 105 bp fragments for the A allele and 222, 105 and 106 bp fragments for the G allele. (**C**) Partial sequence chromatograms of GSTP1 A313G polymorphism (arrow marked) are shown from three subjects whose RFLP data are depicted in B. (D) 3D structure of wild GSTP1 I105 (Camel colour) and mutant GSTP1 V105 (Carolina blue colour). Altered sites were marked by arrows and showed the amino acids and its position.

The modelled mutant protein with amino acid substitution is subjected to energy minimizations and it is observed that the total energy of the mutant proteins is higher than the wild protein. Stereochemical and main chain parameter of validated modelled protein is shown in Supplementary Table S4(a,b). 99.5% (96.1% in most favoured regions) of the residues for mutant GSTP1 lie in allowed regions as revealed by Ramachandran plot indicating the reliability of the structures predicted. Comparative structure analyses of wild and mutant proteins (Fig. 1D,E) reveals the occurrence of secondary structure and protein folding alteration due to single amino acid polymorphism. Gln24, Cys169, Leu170, Asp171, Ala172 and Ala185 have resulted in conversion of loop to helix due to replacement of isoleucine to valine at position 105.

### Protein- protein docking and molecular dynamics simulation of wild and mutant GSTP1- JNK complex

Protein-protein interaction between JNK with wild-type GSTP1 showed high affinity (−850.18 Kcal/mol) than mutant GSTP1 (−757.79) Table 3. Using High Ambiguity Driven protein-protein Docking (HADDOCK), which provides full flexibility to protein side chain, we found a similar affinity (−843.21 Kcal/mol). Our results indicate that H-bond, van der Wall and Cation pi interaction play important role in GSTP1-JNK interaction. GSTP1 AA-genotype had high binding affinity with JNK with 104 H-bond interaction compared to mutant GSTP1-JNK which shows only 78 H-bond interaction. The residues involved in H-bond interactions are summarised in Table 3. Both wild and mutant complex were found to have formed cation pi interaction/s. The interaction between Tyr 50 of wild GSTP1 and JNK has two pi cation interactions whereas only one pi cation interaction showed between mutant GSTP1 and JNK.

**Table 3:** List of residues of native and mutant protein involve in H-bond interactions with JNK.

Docked complex	Hex score K Cal/Mol	No of H-bond	Interacting residue of GSTP1 involve in H-bond		
			MC-MC H-bond	MC-SC H-bond	SC-SC H-bond
Native complex (GSTP1105I-JNK)	−850.18	104	Nil	Cys47, Leu48, Tyr49, Gln64, Met91,Asp94, Asp98, Cys101, Lys102,Tyr108, Thr109, Asn110	Gln39, Tyr49, Gln51,Asn66, Met91,Glu97, Lys102,Thr109, Asp117
Mutant complex (GSTP1 105V-JNK)	−757.79	78	Gln83	Leu48, Tyr49, Thr67, Arg74, Gln83, Asp90, Met91, Asp94, Asp98, Lys102, Thr109, Gly 114, Lys120	Gln51, Gln64, Gln83, Met91, Asn93, Asp94, Asp98, Gln125

MC-MC H-bond = Main Chain-Main Chain Hydrogen Bonds

MC-SC H-bond = Main Chain-Side Chain Hydrogen Bonds

SC-SC H-bond = Side Chain-Side Chain Hydrogen Bond

Analysis of various trajectories of molecular dynamic simulation revealed that the wild complex reached stability compared to mutant at very early phase of simulation which could lead to a weaker binding affinity of mutant GSTP1 to JNK. For detail results, please see the Supplemental Information (Fig. S1).

### Lower c-Jun phosphorylation in normal oral tissues of GSTP1 AA-genotype

**Figure 2:**
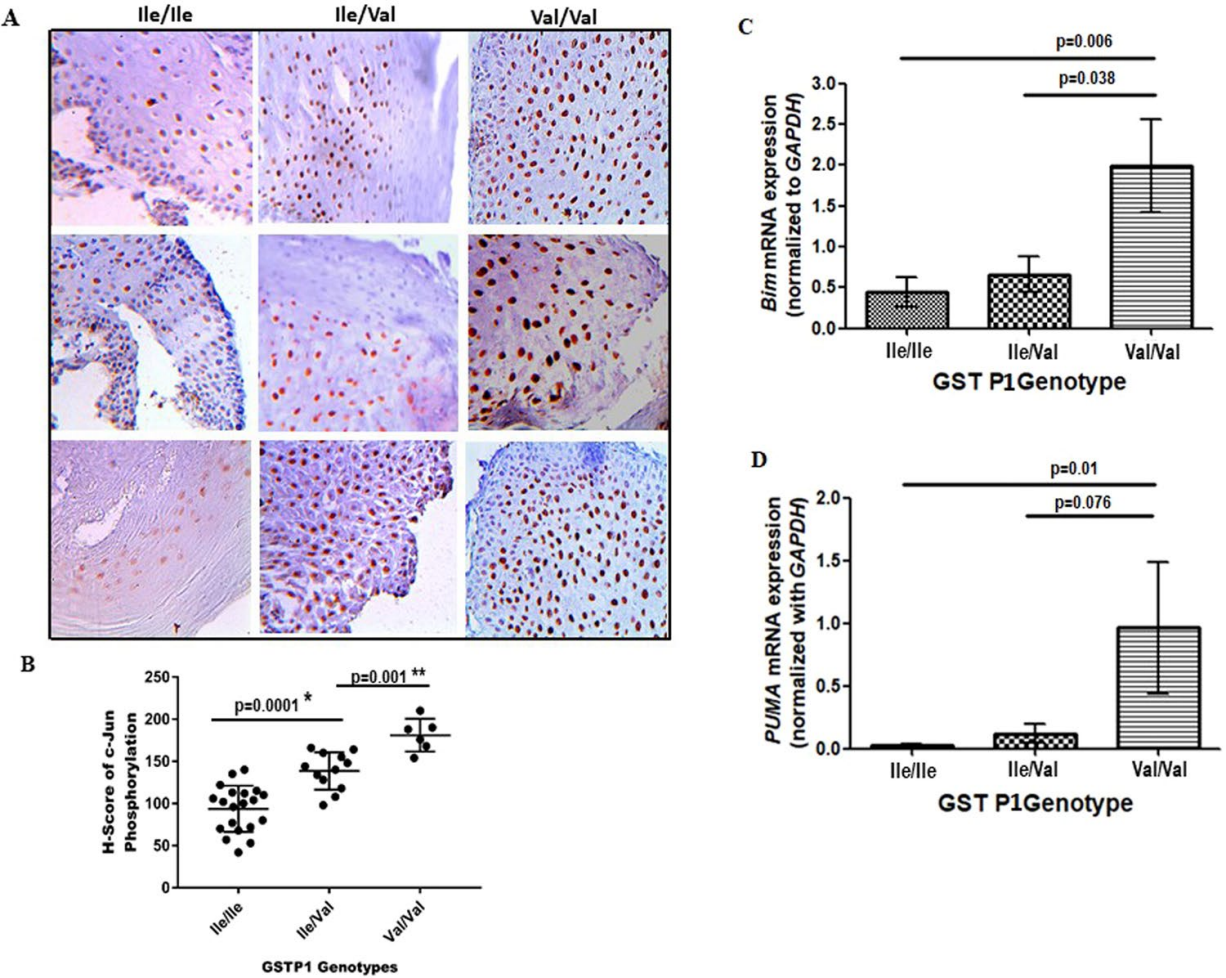
Effect of GSTP1 I105V polymorphism on c-Jun phosphorylation and expression of proapoptotic genes in normal oral tissue in OSCC patients. (A) Representative images of an immunohistochemical analysis of adjacent normal oral tissues in OSCC patients done with anti-c-Jun phosphorylation antibody. Human samples were collected from the persons whose GSTP1 proteins having Ile/Ile or Ile/Val or Val/Val at 105 positions. (**B**) The level of c-Jun phosphorylation in normal oral tissues analyzed by H-score were shown. Data were analyzed by Student’s t-test. *Two-tailed p < 0.004, n = 20 for Ile/Ile and 12 for Ile/Val samples; **P < 0.0001, n = 10 for Ile/Ile and 6 for Val/Val samples. The magnification of all these images is 40X. (**C,D**) Effect of GSTP1 genotypes on quatitative expression of Bim and PUMA mRNA. Total RNA was isolated from tumor cells collected from samples having either GSTP1 Ile/Ile or Ile/Val or Val/Val genotypes for real-time qPCR using either Bim primers (C) or PUMA primers (D) following the procedures mentioned in the Methods. Data are plotted as a histogram. Each bar is the mean ± SD derived from 20 Ile/Ile, 12 Ile/Val and 6 Val/Val samples. The value of p < 0.05 consider to be Significant in unpaired t-test.

We studied c-Jun phosphorylation in a panel of normal oral tissue samples with GSTP1 AA-genotype (Ile/Ile) (n = 20), AV-genotype (Ile/Val) (n = 12) and VV-genotype (Val/Val) (n = 6). Both Ile/Val and Val/Val showed significantly higher c-Jun phosphorylation than Ile/Ile samples (Fig. 2A). H-score (Fig. 2B) of c-Jun phosphorylation varied from 42 to 140 for normal oral tissues with AA-genotype, 108 to 164 for AV-genotype and 154 to 210 for VV-genotype.

### Higher expression of *Bim* and *PUMA* in tumor cells with GSTP1 GG-genotype

The above data indicate that wild GSTP1 has stronger affinity to JNK as compared to mutant GSTP1 and therefore the wild GSTP1 showed lower c-Jun phosphorylation. This might lead to reduce apoptosis by downregulating the proapoptotic genes. We explored this by analysing the expression pattern of *Bim* and *PUMA*, the downstream effectors of the Bcl-2 family. The present data demonstrated that the expression of both *Bim* and *PUMA* were significantly higher in the tumor samples with GSTP1 GG-genotype than the samples with AA-genotype (p value 0.006 for *Bim* and 0.01 for *PUMA*) (Fig. 2C,D).

### Better detoxification of toxins by mutant protein: docking studies

**Figure 3:**
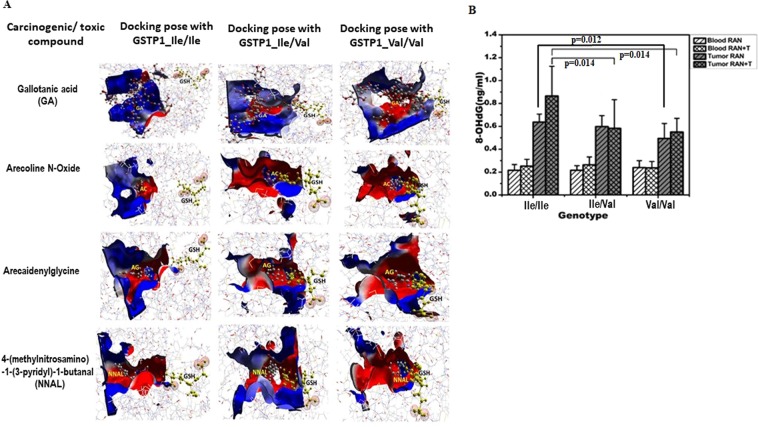
An electrostatic interactions with toxic substances at active site cavity of GSTP1 and its influence on the individual sensitivity to genotoxic effects. (**A**) Comparative electrostatic interactions of reduced GSH and toxic metabolites like, GA, arecoline N-oxide and arecaidenylglycine derived from raw areca-nut, and NNAL derived from tobacco, with dimeric GSTP1 proteins having Ile/Ile, Ile/Val and Val/Val at 105 positions. Red colour indicates negative charge and blue positive charge. The distance of GSH from the active pocket indicates its relative affinity for the active site residues. (**B**) Quantitation of 8-OHdG (ng/ml) in DNA digests was performed by ELISA-kit in both blood and tumor DNA from oral cancer patients having either RAN-chewing or RAN + tobacco-using habit. Data are plotted as a histogram. Each bar is the mean ± SD derived from 12 Ile/Ile, 12 Ile/Val and 6 Val/Val samples. The value of p < 0.05 consider to be Significant in unpaired t-test.

Through docking studies we were able to display the binding ability of wild-type and mutant proteins with GSH and various carcinogens/toxins derived from areca nut/tobacco (four such compounds showed in Fig. 3A and six more showed in supplementary section Fig. S2). Several amino acids of GSTP1 are found to be actively involved in binding of caricinogens/toxins (Table 4 and Supplementary Tables S5 and S6). On the basis of the gold score, it is noted that most of the carcinogens/toxins bind almost equally with both wild and mutant GSTP1, except for gallotanic acid, a derivative of RAN, showed better binding with GSTP1 GG-genotype (Table 4), whereas N’-nitrosonornicotine (NNN), a derivative of tobacco, showed better binding with GSTP1 AA-genotype (Table S6). For effective detoxification process, GSTP1 has to bring GSH and substrate into close proximity inside the binding pocket for conjugation reaction. Hence, presence of both GSH and substrate in binding pocket is crucial for their conjugation. Therefore, further analysis of docking poses of both wild and mutant protein was performed. It was observed that in case of wild-type protein, GSH was at a fair distance from the docking pocket where carcinogenic ligands were bound. In contrast, homodimeric and heterodimeric mutant protein-ligand complexes indicate that both GSH and ligands reside together in the active site pocket of the protein (Fig. 3A). Similar feature was observed with several other toxins derived from either RAN or tobacco (Fig. S2; Table S5 and S6).

**Table 4:** Docking result of wild and mutant GSTP1 with Areca nut/Tobacco carcinogens/toxins.

Carcinogens/metabolites	GSTP1_Ile/Ile		GSTP1_Ile/Val		GSTP1_Val/Val	
	Gold Score	Residue involve in interaction	Gold Score	Residue involve in interaction	Gold Score	Residue involve in interaction
Gallotanic acid	107.15	Gln51(B), Ser105(A), Asn110(A), Gln125(A)	118.46	Tyr7(A), Arg13(A), Gln51(A), Leu52(A), Gln64(A), Asn66(A), Asp94(B), Asp98(B)	121.76	Tyr7(B), Arg13(B), Arg70(A,B), Asp98(A,B)
Arecoline N-Oxide	47.4	Pro53(B), Gln51(B), Gln64(B)	45.21	Asn66(A), Arg70(A,B), Asp94(A)	43.38	Asn66(A), Arg70(A,B), Asp94(B)
Arecaidinyl glycine	40.90	Gln64(B), Ser65(B), Asn66(B), Asp98(A)	39.36	Arg70(A), Asn93(A), Asp94(A,B), Glu97(B), Asp94(A)	38.90	Arg70(A,B), Asn93(A), Asp94(A), Glu97(B), Asp98(B)
4(methylnitrosamino)-1-(3-pyridyl)-1-butanal (NNA)	54.67	Arg13(B), Gln64(B), Ser65(B), Arg70(B), Asn93(B), Asp94(B), Asp98(B)	45.99	Glu97(B), Asp98(B), Cys101(B), Lys102(B)	49.70	Gln64(B), Asn66(B), Ser65(B), Arg70(B), Glu97(B),Asp98(A)

### Higher level of oxidative damages to DNA in tumor cells with GSTP1 AA-genotype

An enzyme-linked immunosorbent assay (ELISA) method was used to measure 8-OHdG, a marker of oxidative damage to DNA, in DNA extracted either from blood lymphocytes or tumor tissues of oral cancer patients who had the habit of RAN-chewing with and without tobacco (Fig. 3B). It was clear that higher level of 8-OHdG was present in DNA in tumor than blood lymphocytes. The level of 8-OHdG in the blood-lymphocytes was similar irrespective to GSTP1 genotypes. In contrast, the level of 8-OHdG in tumor DNA was significantly higher in GSTP1 AA- than GG-genotypes (p value 0.012 in RAN samples; p value 0.014 in RAN + Tobacco samples).

## Discussion

The present study confirms that the AA homozygous genotype of GSTP1 gene is significantly associated with the risk of oral cancer even after adjusting for age, gender and habit of RAN/tobacco usage in the population. The similar results are obtained when tested with the habit-matched case-control data for the two most significant habits “RAN only” and “RAN + Tobacco” separately. GSTP1 AA reference allele is associated with an increased risk for esophageal cancer in those having smoking habit^21^ and laryngeal cancer in those having smoking and drinking habit^31^. Earlier study with Indian samples has documented association of AA-genotype with the risk of oral leukoplakia which is essentially consistent with smokeless tobacco users^32^.

Comparative analysis of wild and mutant GSTP1 protein structure shows the changes in secondary conformation. An earlier investigation on this polymorphism has revealed differences in their thermal stability as well as its specific activity and affinity for electrophilic substrates^18^. Here we have compared the functional efficiency of both GSTP1 wild-type and the mutant in human samples. GSTP1 can regulate activities of several cellular proteins by forming protein:protein interactions with critical kinases involved in controlling stress response, apoptosis and proliferation^33^. The present protein-protein interaction study reveals that due to this polymorphism, binding geometry between GSTP1 GG-genotype and JNK is disrupted which weakens the affinity between these two proteins. The molecular dynamic simulation study indicates that the GSTP1 AA-genotype reaches stability at very early phase of simulation. Additionally, lower energy value of “wild complex” compared with the “mutant complex” indicates greater stability of the former. Therefore, present result indicates that GSTP1 AA-genotype has stronger affinity to JNK as compared with the mutant which might impair c-Jun-phosphorylation and reduce the extent of apoptosis. We have now validated it experimentally in normal human oral epithelial cells in this study. This assumption is further strengthened after observing an increased expression of pro-apoptotic proteins *Bim* and *Puma*, the downstream effector of the Bcl-2 family, in the tumor samples having GG- rather than AA-genotype. Both *Bim and Puma*, proapoptotic proteins are transcriptionally activated by JNK/cJUN axis^34^. It was earlier demonstrated in cell lines that the binding of GSTP1 to JNK1 is a crucial step in apoptosis repression^33,35^. Higher activity of JNK and consequent phosphorylation of c-Jun was observed in mice without GSTP1^36^.

The active site of GSTP1 consists of a hydrophilic G-site (glutathione [GSH]-binding site) and a hydrophobic H-site (xenobiotic-binding site)^37^. Since the data on association between different metabolites of RAN/tobacco and GSTP1 polymorphisms are scarce, we adopted an *in silico* approach to assess the association of GSTP1 Ile/Ile with the susceptibility to RAN/tobacco metabolites than that of GSTP1 Ile/Val or Val/Val. Comparative electrostatic interaction of reduced GSH with RAN-derived toxic compounds such as N-methylnipecotyl-glycine (NMNG) and arecaidine and two other tobacco-derived toxic compounds was shown recently^12^. In this study, similar interaction between other toxic metabolites of RAN/tobacco and reduced GSH was evaluated *in silico*. It showed that Val105 substitution results in steric restriction of the H-site due to shifts in the side chains of several amino acids leading to reduce the distance between G-site and H-site whereas in the Ile/Ile form such distance is increased suggesting less detoxification. Earlier it has been reported that the structure of GSTP1 Val, has more surrounding water molecules which are linked to a channel of additional water molecules in contrast to GSTP1 Ile, which is proposed to influence the catalytic process^38^. It has earlier been demonstrated that GSTP1 Val increases catalytic efficiency by several fold towards tobacco-related pollutants benzo(a)pyrene, and diol epoxide as compared to GSTP1 wild type enzyme^39^. Thus, weak detoxification of the RAN/tobacco metabolites by GSTP1 Ile/Ile leads to higher induction of oxidative damages to DNA leading to mutagenesis and genomic instability. The present quantification of 8-OHdG has been widely used earlier, not only as a biomarker indicating the level of endogenous oxidative damage to DNA but also as a risk factor for several diseases, including cancer^40^. Higher level of 8-OHdG has been noted in smokers than non-smokers^30^. It has been demonstrated that reactive oxygen species mediated DNA double strand breaks and 8-OHdG occurs via secretory cytokines in areca-nut exposed oral keratinocytes^41^. With this rational in view, validation of the present *in silico* observations was sought to be done by quantifying the level of 8-OHdG in DNA digests of cancer cells obtained from patients with different GSTP1 genotypes. Presence of significantly higher level of 8-OHdG in cancer DNA samples with AA-genotypes suggested that metabolic activation of RAN/tobacco in the oral cavity could produce a variety of toxic substances which induce various damages^42,43^ including 8-OHdG^44^. This could be the reason for higher 8-OHdG observed in tumor DNA than DNA from peripheral blood of the same patient. Thus, metabolic activation and detoxification are considered to be an important factor in determining the ultimate effects of exposure to chemical carcinogens.

## Conclusions

The GSTP1 AA reference allele (rs1695) is significantly associated with the risk of oral cancer to those having RAN consumption habit with and without tobacco. Such association can be attributed due to poor detoxification of RAN/tobacco toxins and lowering c-Jun phosphor-ylation due to its strong binding to JNK which consequently may inhibit apoptosis. Thus, it can be said that the development of cancer is not only due to the type of habit that patients have but depends on interaction between the metabolites and the genes that detoxify these metabolites/carcinogens. Nowadays, SNPs have been considered as more tractable genotypic markers^45^ and can be utilized in human genetic analysis which can provide critical proof-of-concept of a priori prediction of responses to certain food habit and environmental exposure. These data also provide a foundation for future genotype-phenotype association studies involving carcinogenesis risk.

## Materials and Methods

### Selection of study participants

The samples for the present study was collected from Nazareth hospital, Shillong, India. A total of 445 Oral Cancer patients and 444 healthy controls were recruited and peripheral blood sample was collected from each donor in heparinized vials, under aseptic condition. Of the total 445 oral cancer patients, 192 were only RAN chewers and 253 were from both RAN and tobacco chewing category. The age ranged from 28 to 84 years (mean ± SD; 53.8 ± 12.0) for oral cancer samples whereas the age varied from 21 to 90 years (mean ± SD; 45.4 ± 17.7) for healthy control group. For the details about demographic characteristics of the samples, please see the Supplemental Information Table S1. All the donors had no viral diseases or antibiotic therapy during the last 6 months. This study was approved by the Institutional Ethics Committee for Human Samples/Participants (IECHSP/2014/07) in North-Eastern Hill University, Shillong, India. The tumor tissues were obtained from patients after having their consent for participation and were individually interviewed before taking the biopsy. Informed consent was obtained from all the individuals studied before sample collection. Every biopsy was kept in RNAlater soon after its collection and all experiments were performed in accordance with relevant guidelines and regulations.

A total of 38 normal oral squamous cell epithelium (3cms away from the cancer site) were also collected from oral cancer patients. Biopsy and resection samples were reviewed by the pathologists and Head and Neck Surgery Department of Nazareth Hospital to confirm the diagnoses and also select representative blocks for immunohistochemical analyses.

### DNA isolation and SNP genotyping

Genomic DNA was extracted from 3 ml peripheral blood using proteinase K treatment and the standard phenol-chloroform extraction procedure^46^. The details of primer sequences (Table S2) and about the PCR reaction are given in the Supplementary section.

The genetic polymorphism of GSTP1 in exon 5 (rs1695, Ile/Ile, Ile/Val, Val/Val genotypes) was identified using the *Alw26*I restriction enzyme^47^. A 433 bp fragment of GSTP1 gene was amplified and the presence of Alw26I restriction site yielded 328 and 105 bp fragments, respectively (GSTP1 Ile/Ile). The presence of rs1695 (313 A/G) creates another restriction site within the 328 bp fragment which when digested by Alw26I, yielded two fragments of 222 and 106 bp (GSTP1 Val/Val) (Fig. 1A,B).

To validate the genotype data generated by PCR-RFLP for rs1695, a subset of samples (100 in each) were resequenced for the GSTP1 gene using the same primer pairs that were used for PCR during the RFLP assay. The sequencing reactions were performed by conventional Sanger sequencing method using an ABI PRISM 3100 Genetic Analyzer and the genotypes were determined from the electropherograms using Seqscape v.2.4 (Applied Biosystems) (Fig. 1C).

### Statistical analysis

Estimation of allele and genotype frequencies for both the SNPs in the cases and controls and tests for deviation from Hardy-Weinberg equilibrium on the control group were performed using SPSS 20.0 and GraphPad Prism software, respectively. Case-control association study was performed for rs1695 to find out the risk genotype, if any, associated with the risk of developing oral cancer in Meghalaya, India. For comparing genotype frequencies of GSTP1 between cases and controls, the individuals were grouped into reference allele homozygous (AA) and mutant allele containing (AG + GG) genotypic groups, p-value estimation was done by Chi-square tests or Fisher exact tests used appropriately. For adjusting the influence of confounding covariates like age, gender and habit on association of risk genotypes with oral cancer, multivariate binary logistic regression was performed to compute Odds Ratio and 95% C.I. with case-control affection status as dependent variable and SNP genotypes (AA vs AG + GG), age, sex and habit as independent variables using SPSS 20.0. Habit-matched case-control analysis was performed for both the “Raw Areca-Nut only” and “Raw Areca- Nut+Tobacco” groups to understand the independent association of SNP genotypes with the risk of development of oral cancer compared to controls when the influence of habit is not a probable confounder.

### Immunohistochemistry (IHC)

A total of 38 normal oral squamous cell were classified according to their genotypes of GSTP1 having Ile/Ile, Ile/Val and Val/Val at 105 positions. These normal tissue samples were dehydrated, paraffin embedded and sectioned with a microtome (Leica). Briefly, after blocking for endogenous peroxidase activity, the sections were incubated with anti-c-Jun phosphorylation (ab32385; Abcam, USA) primary antibody. IHC analysis was performed with a Strept-Avidin Biotin Kit (Dako). The scoring of immunohistochemical stains in each specimen was determined using a histological score (H)^48^. The H-score is computed on the basis of both extent and intensity of staining on the scale of 0, 1, 2 and 3, representing negative, weak, moderate and strong staining. Finally, the H-score had been obtained by multiplying the staining intensity by the percentage of positive cytoplasmic staining cells (Supplemental Information).

### Quantitative real-time PCR

Total RNA was isolated with Trizol from tumor as well as normal tissue samples collected from each patient and then purified using the RNeasy Mini Kit (Qiagen) according to the manufacturer’s protocol. Synthesis of cDNA was performed with 1 μg of total RNA from each sample using Quantiscript Reverse Transcriptase, Quantiscript RT-buffer and RT Primenr-mix of QuantiTect Reverse Transcription kit (Qiagen GmbH, Hilden, Germany) according to the manufacturer’s protocol. Quantitative real-time PCR was performed using in 96-well optical reaction plates (Applied Biosystems, Darmstadt, Germany) using a StepOnePlus amplification and detection system (Applied Biosystems). The real-time RT-qPCR reactions were prepared using SYBR® Select Master Mix (Life Technologies), and the following conditions were used: 95 °C for 5 min, 40 cycles of 95 °C for 30 s, 60 °C for 30 s and 72 °C for 30 s. The primers of target genes used for this analysis were Bim and PUMA, and GAPDH was used as the reference gene. The primer sequences are listed in Table S3. The gene copy numbers of *Bim* and *PUMA* were calculated by using a standard curve that was constructed using the OE33 cell line. The 2^−ΔΔ*CT*^ method was used as a relative quantification strategy for qPCR data analysis. In total, 20 samples from Ile/Ile, 12 from Ile/Val and 6 from Val/Val genotype were used in this study.

### 8-OHdG measurement

Measurement of 8-Hydroxydeoxyguanosine (8-OHdG), a known marker of oxidative stress-mediated DNA damage, was estimated with OxiSelect^™^ Oxidative DNA damage ELISA-kit, Cell Biolabs Inc. (San Diego, CA) in DNA of blood lymphocytes and tumor tissues from the cancer patients having the habit of chewing RAN with and without tobacco. DNA were extracted from blood and tumor tissue of the same patient (12 from Ile/Ile, 12 from Ile/Val and 6 from Val/Val) and digested with nuclease P1 and calf intestinal phosphatase (Sigma, USA) and denatured. 8-OHdG was quantified by quantitative ELISA assay in 96-well plate format. The quantity of 8-OHdG in the specimens were determined by comparing its absorbance with known 8-OHdG standard curve.

### Mutant protein modelling and structure validation

A graphical program for computational aided protein engineering, TRITON has been used for modelling GSTP1 protein mutant^49^ and energy minimization for 3D structures was performed with GROMOS 4.5.4 package using OPLS (Optimised Potential for liquid simulation) force field^50^. The functional form of the OPLS force filled is very similar to that of the Amber and is represented by

$${\rm{E}}\,{({\rm{r}}}^{{\rm{N}}})={{\rm{E}}}_{{\rm{bonds}}}+{{\rm{E}}}_{{\rm{angles}}}+{{\rm{E}}}_{{\rm{dihedrals}}}+{{\rm{E}}}_{{\rm{nonbonded}}}$$

### Protein-protein docking

In this study, the docking analysis of JNK with wild and mutant GSTP1 was initiated using Hex8.0.0 program^51^ for automated comparative protein–protein docking. In this study JNK was treated as receptor, while GSTP1 wild and GSTP1 mutated proteins were treated as ligand.

Further, Protein-protein docking was also carried with HADDOCK tool since it provides full flexibility to protein side chains. HADDOCK is an information-driven flexible docking approach for the modelling of biomolecular complexes.

### Molecular dynamic simulation of wild and mutant complex

To study the dynamic behaviour of the protein, molecular dynamic simulation of both wild and mutant protein complex was performed. The docked complexes of JNK with native and mutant GSTP1 generated by Hex were used as a starting point for molecular dynamic simulation which was carried out with GROMACS 4.5.4 package using OPLS force field. The details methodology is mentioned in the Supplemental Information.

## Comparative catalytic activity of wild and mutant protein by docking studies

### Protein and ligand preparation

X-ray crystallographic structure of wild protein (19GS) was obtained from protein data bank, and the mutant protein was modeled using TRITON. GSTs bind and detoxify substrate in their dimeric form and therefore only protein dimers have been selected in this study. The carcinogenic/toxic compounds from areca nut and tobacco have been used to study their binding affinity for homodimeric wild (GSTP1 Ile/Ile), homodimeric mutant (GSTP1 Val/Val) and heterodimeric mutant (GSTP1 Ile/Val) proteins. CASTp server^52^ was used for the identification of active site of the proteins. The first step of catalytic mechanism is the interaction of GSTs with GSH to activate it for nucleophilic attach. Hence both wild and mutant proteins were first docked with GSH and best docked complex has been chosen as basic protein structure for further docking studies with areca nut and tobacco derived xenobiotics.

### Protein-ligand docking studies

*In silico* docking approach was used to study the binding affinity of 22 compounds reported from areca nut and tobacco^28,29^ with wild and mutant GSTP1 by using GOLD v5.2 software. GOLD is genetic algorithm (GA) based docking program. The algorithm allows full flexibility of the ligand and partial flexibility of the protein. The best and most energetically favourable conformation of each compound was selected. GOLD gives the binding result in term of GOLD score or Fitness score. GOLD fitness function is made up of four components: Protein-ligand hydrogen bond energy, Protein-ligand van der Waals energy, ligand internal van der Waals energy and ligand torsional strain energy.

## Supplementary information

Supplementary information.

## Data Availability

The data that generated and supports the findings of this study will be available by the corresponding author upon request.
