# Supplementary material for: Glutathione S-transferasesP1 AA (105Ile) allele increases oral cancer risk, interacts strongly with c-Jun Kinase and weakly detoxifies areca-nut metabolites
Source: Sci Rep. 2020 Apr 7;10:6032. doi: 10.1038/s41598-020-63034-3 (PMC7138809; doi:10.1038/s41598-020-63034-3)
Supplement: Supplementary file 1 — Supplementary information. [file 41598_2020_63034_MOESM1_ESM.docx]

**Supplementary Section**

**Glutathione S-transferasesP1 AA (105Ile) allele increases oral cancer risk, interacts strongly with c-Jun Kinase and weakly detoxifies areca-nut metabolites**

**Pallavi Yadav*, Atanu Banerjee*, Nabamita Boruah, Chongtham Sovachandra Singh, Puja Chatterjee^1^, Souvik Mukherjee^2^, Hughbert Dakhar^3^, Henry B Nongrum^4^, Atanu Bhattacharjee, Anupam Chatterjee^#^**

Molecular Genetics Laboratory, Department of Biotechnology & Bioinformatics, North-Eastern Hill University, Shillong, Meghalaya, India-793022, **^1^** Oral and Maxiofacial Surgery, MR Ambedkar Dental College and Hospital, Cooke Town, Bengalore-560004, Karnataka, India; **^2^**National Institute of Biomedical Genomics, Kalyani-741251, West Bengal, India **^3^**Histopathology Division, **^4^**Otolaryngology and Head and Neck Surgery Department, Nazareth Hospital, Laitumkhrah, Shillong-793003, India.

* Equal contribution

**^#^Corresponding Author**: Anupam Chatterjee, Department of Biotechnology & Bioinformatics, North-Eastern Hill University, Shillong, Meghalaya-793022, India.

Tel: +91 364 2722403; Email: [anupamchatterjee@nehu.ac.in](mailto:anupamchatterjee@nehu.ac.in); [chatterjeeanupam@hotmail.com](mailto:chatterjeeanupam@hotmail.com)

**Material and Methods**

**Supplementary Table**

**Table S1**  Demographic Characteristics of Study Populations

**Characteristics Healthy Control Group Oral Cancer Patients**

**(n=444) (n=445)**

**Age (Years)**

Mean ±SD 45.4 ± 17.7 53.8 ± 12.0

**Sex**

Male 216 239

Female 228 206

**Habit**

Nil 72 00

Areca Nut 162 192

Areca nut + Tobacco 210 253

**Table S2** The sequence of primer pairs used in PCR reaction.

**Gene name Primer Primer sequence Product size**

**GSTP1 GSTP1F 5’-GTAGTTTGCCCAAGGTCAAG-3’ 433bp**

**GSTP1R 5’-AGCCACCTGAGGGGTAAG-3’**

**PCR Reaction**

The composition of the PCR reaction mixture consisted of 100 ng genomic DNA, 1×PCR buffer containing 1.5 mM MgCl_2_, 0.2 mM dNTP mixture, 0.2 μM of both primer sets and 1 U Taq DNA polymerase (Invitrogen) in final volume of 50 μl. The PCR conditions for both reactions were as follows: denaturation step at 95°C for 3 minutes, followed by 30 cycles of denaturation at 95°C for 30 seconds, annealing at 63°C for 30 seconds, elongation at 72°C for 30 seconds and a final extension at 72°C for 7 minutes. PCR products for rs1695 polymorphism**s** were subjected to enzymatic digestion by incubation with *Alw26I* enzymes respectively for 5 hr at 37°C and then electrophoresis was performed using 2% agarose gels.

**Immunohistochemical analysis**

Immunohistochemical staining was performed on formalin fixed, paraffin embedded tissues that were sectioned at 5 µm thickness. Slides were deparaffinized, rehydrated and treated with 3% H_2_O_2_ in PBS. Antigen retrieval was done with 0.01% sodium-citrate buffer followed by blocking in PBST containing 0.1% BSA and 10% FBS. Primary antibody incubation was carried out for overnight. Slides were washed and incubated with appropriate biotinylated secondary antibody for 1 hr at room temperature. Following washing, slides were treated with streptavidin-HRP (1:1000) and subsequently washed (PBS containing 0.1% Tween-20) and color was developed with DAB+ H_2_O_2_. Slides were counterstained with haematoxylin, washed and mounted in DPX (Sigma-Aldrich, USA). The staining intensity of the images was evaluated by two independent investigators.

In the regions of the histologic sections, the chromogenic immunolabeling was systematically categorized into four groups: 0 (no labelling), 1+ (weak labelling), 2+ (moderate labelling), and 3+ (strong staining; observable with 10x objective). Semi-quantitative staining analysis was done by H-score by counting 500 cells from five different fields in the slides considering low, moderate and higher intensity of c-Jun phosphorylation. The percentage of positive cells with a given intensity for each sample was determined independently by a pathologist and a trained reader. A single manual H-score based on a scale of 0 to 250 was generated for each labelled section by taking the sum of the percentage of cells labelling 1+, double the percentage of cells labelling 2+, and triple the percentage of cells labelling 3+ (H-Score= ((%3+) × 3) + ((%2+) × 2) + (%1+)). Student’s t-test was performed for comparing the c-Jun phosphorylation in normal samples having different GSTP1 genotypes at 105 position. Statistical significance was considered when p-value is less than 0.05.

**Table S3** The sequence of primer pairs used in quantitative real-time PCR reaction.

**Gene name Primer Primer sequence Product size**

Bim Bim (F) 5’–TATGAGAAGATCCTCCCTGC–3’ 180bp

Bim (R) 5’–ATATCTGCAGGTTCAGCCTG–3’

PUMA PUMA (F) 5’–CCTGGAGGGTCCTGTACAATCT–3’ 88bp

PUMA (R) 5’–GCACCTAATTGGGCTCCATCT–3’

GAPDH GAPDH (F) 5’–ACAGTCCATGCCATCACTGCC–3’ 265bp

GAPDH (R) 5’–GCCTGCTTCACCACCTTCTTG–3’

**Molecular dynamic (MD) simulation of wild and mutant complex**

To study the dynamic behaviour of the protein, molecular dynamic simulation of both wild and mutant protein complex of 20 ns was performed. The complexes were solvated in a cubic box of simple point charge water molecules. Complexes were found to be negatively charged at physiological pH therefore six Na^+^ counter ions were added by replacing water molecules to make the system electrically neutral. Native and mutant complex were subjected to energy minimization for 50,000 steps by steepest descent algorithm. In order to equilibrate the system the solute was subjected to position-restrained dynamics simulation at 300 K for 100 ps. Berendsen temperature coupling method was used to regulate temperature inside the box. Electrostatic interactions were computed using the particle Mesh Ewald method. Finally, the system was subjected to MD simulation at 300 K temperature and 1 bar pressure for 20,000 ps. We then compute the comparative analysis of structural deviations in native and mutant complex. Analysis of trajectories root mean square deviation (RMSD), solvent accessible surface area (SASA), Radius of gyration (Rg) and energy) were carried out using XMGRACE program.

**Results**

**Table S4 (a)** Stereochemical properties of modelled protein

| Stereochemical parameters | GSTP1_V105 |
| --- | --- |
| Residue in most favoured region (%)  Residue in additional allowed region (%)  Residue in generously allowed region (%)  Residue in disallowed region (%)  Overall G-value  M/C bond length (%)  M/C bond angle (%)  Planar groups (%) | 96.1  3.4  0.0  0.6  -0.34  96.7  85.8  100 |

**Table S4 (b)** Main chain parameters for modeled proteins

| Stereochemical parameters | No. of data Pts | Parameter value | Comparison Typical value | Values Band width | No. of band widths from mean | |
| --- | --- | --- | --- | --- | --- | --- |
| Percentage residues in most favored region | 179 | 96.1 | 88.2 | 10.0 | | 0.8 |
| Omega angle stdev | 206 | 3.9 | 6.0 | 3.0 | | -0.7 |
| Bad contacts/100 residues | 6 | 2.9 | 1.0 | 10.0 | | 0.2 |
| Zeta angle stdev | 190 | 9.1 | 3.1 | 1.6 | | 3.7 |
| H- bond energy stdev | 138 | 0.6 | 0.7 | 0.2 | | -0.4 |
| Overall G- factor | 208 | -0. | -0.2 | 0.3 | | -0.5 |

**Molecular dynamics simulation of wild and mutant GSTP1- JNK complex**

Wild complex showed higher RMSD till 1.3ns and subsequently it reached a steady equilibrium whereas in case of mutant complex it reached equilibrium after 12.55ns (Fig. S1a). RMSD results indicate that the wild complex attains a stable conformation at early phase of simulation. Rg graph tells us about compactness and overall dimension of protein. During the first 2.5 ns higher deviation with increase in Rg score was noticed and thereafter, Rg value was reduced and stabilized at 12 ns for wild complex. In contrast, the mutant complex showed abrupt rise in Rg score till 13 ns (Fig. S1b). Analysis of SASA showed that during first 3 ns wild complex showed a rise in value after which a time dependent decline in SASA was observed (Fig. S1c), whereas in mutant complex, a gradual increase in SASA was noted. However, after 13ns both complexes tend to reach equilibrium. SASA value tells about hydrophobicity/hydrophilicity of protein indicates that some hydrophobic residues tend to be exposed to the outer surface after mutation of GSTP1. This probably reduce the binding affinity of JNK to mutant GSTP1 protein. Higher energy value of mutant complex was observed as compared to wild indicate more stability of wild complex than mutant one (Fig. S1d). Binding free energy for wild complex was -13.5 Kcal/mol whereas for mutant complex it was 3.8 Kcal/mol.

**Table S5** Docking result of wild and mutant GSTP1 with Areca nut carcinogens/toxins

| **S.No.** | **Carcinogens/ metabolites** | **GSTP1_Ile/Ile** | | **GSTP1_Ile/Val** | | **GSTP1_Val/Val** | |
| --- | --- | --- | --- | --- | --- | --- | --- |
|  |  | **Gold Score** | **Residue involve in interaction** | **Gold Score** | **Residue involve in interaction** | **Gold Score** | **Residue involve in interaction** |
| 1 | 3-(methylnitrosamino)- propionitrile (MNPN) | 38.04 | Asn66(A), Arg70(A,B), Asn93(A), Asp94(A,B) | 36.30 | Lys102(B), Asp94(B), Leu99(B), Gln125(B) | 36.6 | Arg70(A,B), Asn93(A), Asp94(A,B) |
| 2 | 3-methylnitrosamino- propionaldehyde (MNPA) | 40.73 | Arg70(A,B), Asn93(B), Asp94(A,B) | 35.81 | Asn66(A,B), Arg70(A,B), Asn93(B), Asp94(A,B) | 37.29 | Asn 66(A,B), Arg70(A,B), Asn93(A), Asp94(A,B) |
| 3 | Arecaidine N-oxide | 37.91 | Gln51(A), Gly95(B), Lys102(B), Gln125(B) | 40.03 | Gln51(A), Gly95(B), Lys102(B), Asp98(B) | 40.9 | Gln64(A), Asn66(A), Asp94(A,B), Asp98(B) |
| **4** | Arecaidine | 32.63 | Arg13(B), Gln64(B), Ser65(B), Asn66(B), Asp94(A), Glu97(B) | 32.40 | Asn66(B), Glu97(A), Asp94(A,B), Asp98(B) | 32.65 | Gln64(A), Asp94(A,B), Asp98(B) |
| 5 | Arecaidinyl glycerol | 54.1 | Arg70(A,B), Asn93(B), Asp94(A,B),  Glu97(A), Asp98(B), Cys101(A) | 45 | Arg70(A,B), Asn93(A,B), Asp94(A,B), Glu97(B), Asp94(A) | 50.6 | Arg70(A,B), Asn93(A), Asp94(A,B),  Glu97(B), Asp98(A) |
| 6 | Arecoline | 47.4 | Pro53(B), Gln51(B), GLN64(B) | 45.21 | Asn66(A), Arg70(A,B), Asp94(B) | 43.38 | Asn66(A), Arg70(A,B), Asp94(B) |
| 7 | Catechol | 34.13 | Asn66(B), Arg70(A,B), Asp94(A,B) | 34.21 | Arg70(A,B), Asn93(B), Asp94(B) | 35.24 | Arg70(A,B), Asp94(A,B), Asn93(A), Glu97(B) |
| 8 | Nitrosoguvacine (NGC) | 40.86 | Asn66(A), Arg70(A), Asn93(A), Asp94(A,B) | 36.21 | Asn66(B), Arg70(A,B), Asn93(B), Asp94(A,B), Glu97(A) | 38.41 | Arg70(A), Asp94(B), Glu97(B), Asp98(B) |
| 9 | Nitrosoguvacoline (NGL) | 48.8 | Asn66(A), Arg70(A,B), Asn93(A,B),  Asp94(A,B), Glu97(B) | 47.04 | Gln64(B), Arg70(A,B), Asp94(A,B), Glu97(B) | 47.69 | Glu64(B), Arg70(A,B),  Asp94(A,B), Glu97(B) |
| 10 | N-methylnipecotyl-glycine (NMNG) | 35.92 | Asn66(A), Arg70(B), Asn93(B),  Asp94(A,B), Glu97(B) | 35.59 | Arg70(A,B), Asn93(A), Asp94(B) | 36.76 | Arg70(A,B), Asn93(A,)  Asp94(B), |

**Table S6** Docking result of wild and mutant GSTP1 with tobacco carcinogens/toxins

| **S.No.** | **Carcinogens** | **GSTP1_Ile/Ile** | | **GSTP1_Ile/Val** | | **GSTP1_Val/Val** | |
| --- | --- | --- | --- | --- | --- | --- | --- |
|  |  | **Gold Score** | **Residue involve in interaction** | **Gold Score** | **Residue involve in interaction** | **Gold Score** | **Residue involve in interaction** |
| **1** | N-Nitrodiethylamine | 31.20 | Gly 95(B), Asp98(B), Leu99(B),Lys102(B), Gln125(B) | 29.14 | Asp98(B), Leu99(B),Lys102(B), Gln125(B) | 30.48 | Arg70(A,B), Asn93(A), Asp94(B) |
| **2** | N-Nitrodimethylamine | 26.55 | Tyr49(B),Gly95(A), Leu99(A), Lys102(A), Gln125(A) | 26.67 | Gly95(B), Asp98(B), Leu99(B),Lys102(B) | 27.84 | Arg70(A,B), Asn93(A), Asp94(B) |
| 3 | 4(methylnitrosamino) -1-(3-pyridyl)-1-butanone (NNK) | 51.23 | Asn66(A), Arg70(A), Asn93(A), Asp94(A,B), Asp98(B) | 53.13 | Asn66(B), Arg70(A,B), Asn93(B), Asp94(A,B), Asp98(A) | 49.31 | Gln64(A), Arg70(A,B), Asn93(A), Asp94(A,B) |
| 4 | Anti-CDE | 58.8 | Asn66(B), Arg70(A,B),  Asp94(A), Glu97(A,B), Cys101(A), Asp98(B) | 43.10 | Glu97(A,B), Asp98(A) | 50.20 | Asn66(A,B), Asn93(A), Asp98(A), Glu97(B) |
| 5 | Benzo[a]pyrene | 51.25 | Asn66(B), Gln64(B),  Asp94(B), Glu97(A,B), Asp98(B) | 45.68 | Tyr7(A), Arg13(A),Tyr108(A) | 46.20 | Arg13(B), Gln64(B), Ser65(B)  Asp98(A), Glu97(B) |
| **6** | N’-nitrosoanabasine | 45.08 | Arg13(B), Gln51(B), Leu52(B),Gln64(B), Ser65(B), Asn66(B), | 40.84 | Asn66(B), Arg70(A), Asn93(A), Asp94(A), Glu97(A) | 39.3 | Gln64(B), Asn66(A), Arg70(A),  Asp94(A,B),Asp98(A), Glu97(B) |
| **7** | N'-nitrosonornicotine (NNN) | 45.07 | Gln51(B), Gln64(B), Asn66(B), Ser65(B), Asp98(A) | 38.72 | Asn66(A), Arg70(A), Asn93(A), Asp94(A), Glu97(A), Asp98(B) | 37.75 | Asn66(B), Asp94(A), Glu97(B), Asp94(A) |
| **8** | N'-nitrosoanabasine (NAB) | 42.77 | Arg13(B), Gln51(B), Leu52(B),Gln64(B), Ser65(B), | 39.86 | Asn66(A), Arg70(A), Asn93(A), Asp94(A), Glu97(A), Asp98(A) | 39.40 | Gln64(B), Arg70(A),  Asp94(A,B),Asp98(A), Glu97(B) |

**Figure Legends**

**Figure S1**. **Molecular dynamic simulation results of GSTP1 and JNK complex.** Structural properties of wild and mutant GSTP1 and JNK complex. **(a)** The Root Mean Square Deviation (RMSD) of the backbone atoms of wild GSTP1 and JNK complex (black colour) and mutant GSTP1 with JNK (red colour) relative to its crystal structure as a function of time is shown. **(b)** The Rg values for wild GSTP1-JNK complex (black color) and mutant GSTP1-JNK complex (red colour) is shown. Lower Rg value indicates maintenance of compactness of protein complexes and overall dimension, which ultimately leads to stability. The wild GSTP1-JNK complex is seen to stabilize with simulation time and reverse is the case with the mutant GSTP1-JNK complex. **(c)** The Solvent Access Surface Area (SASA) analysis of wild GSTP1-JNK complex (black colour) and mutant GSTP1-JNK complex (red colour) is depicted. High SASA values indicates exposure of hydrophobic residues to exterior surface, rendering protein complex unstable. **(d)** The overall energy of the wild GSTP1-JNK complex (black colour) and mutant GSTP1-JNK complex (red colour) is shown. The wild GSTP1-JNK complex is seen to maintain lower energy throughout 20ns simulation time indicating the stability of the complex over the mutated form.

**Figure S2. An electrostatic interaction with toxic substances at active site cavity of GSTP1.** Comparative electrostatic interactions of reduced GSH and different toxic metabolites derived either from raw areca-nut and tobacco, with dimeric GSTP1 proteins having Ile/Ile, Ile/Val and Val/Val at 105 positions. Red colour indicates negative charge and blue positive charge. The distance of GSH from the active pocket indicates its relative affinity for the active site residues.

**Figure S1.** Molecular dynamic simulation results of GSTP1 and JNK complex

**
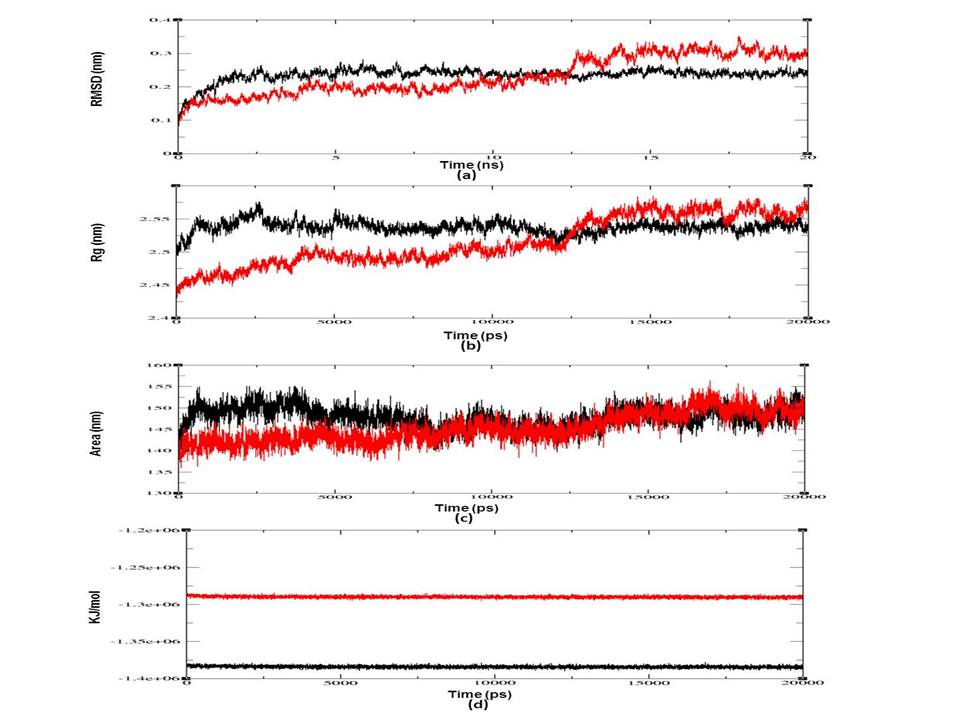
**

| **Figure S2** Comparative docking pose of wild and mutant GSTP1 proteins with carcinogens and GSH at their  ligand binding pocket | | | |
| --- | --- | --- | --- |
| **Carcinogenic/ toxic compound** | **Docking pose with GSTP1_Ile/Ile** | **Docking pose with GSTP1_Ile/Val** | **Docking pose with GSTP1_Val/Val** |
| Arecaidine  (AD) | **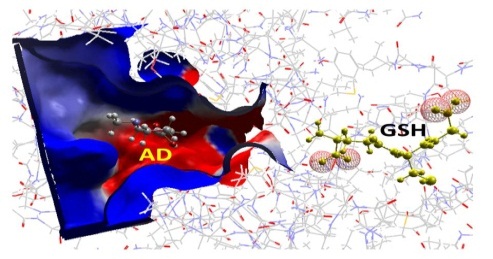** | **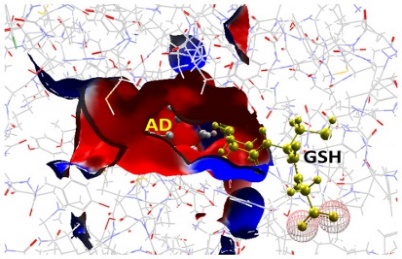** | **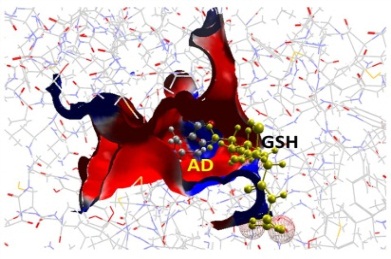** |
| N-Nitrodiethylamine  (NNDE) | **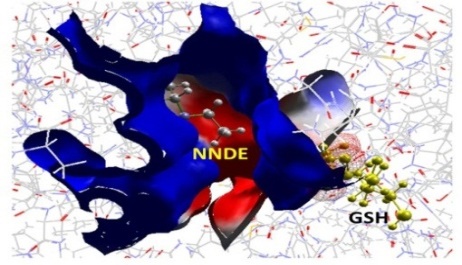** | **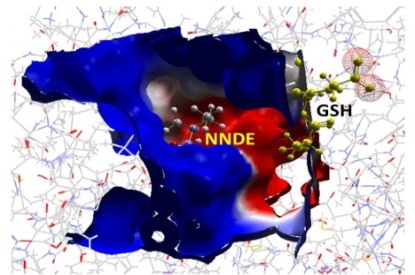** | **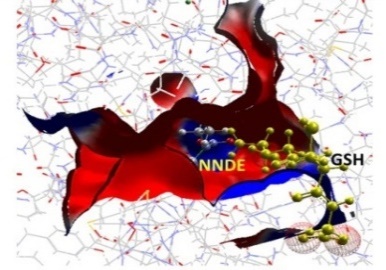** |
| N-Nitrodimethylamine  (NNDM) | **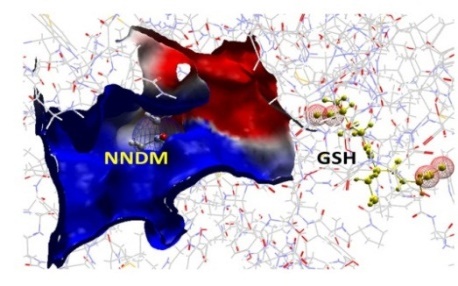** | **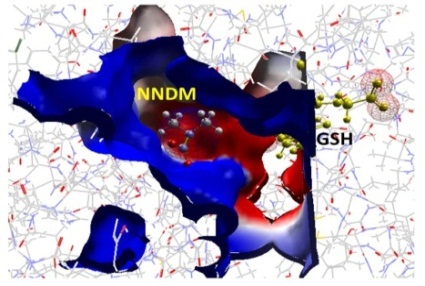** | **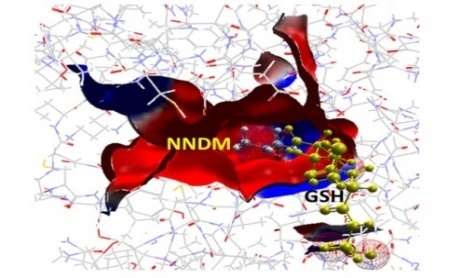** |
| N’-nitrosoanabasine (NAB)  N’-nitrosonor-  nicotine (NNN)  N-methylnipecotyl-  glycine (NMNG**)** | **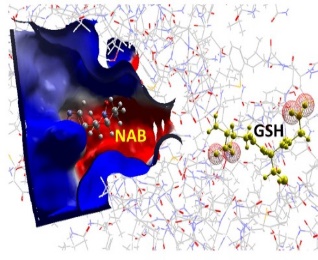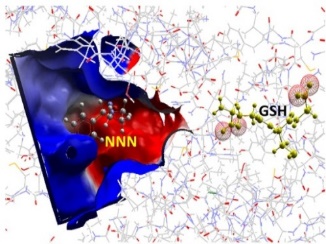**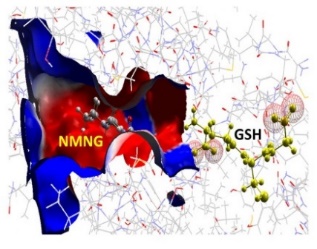 | **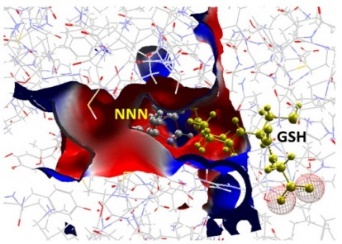**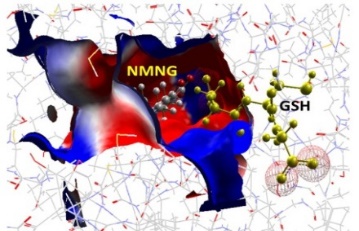 | 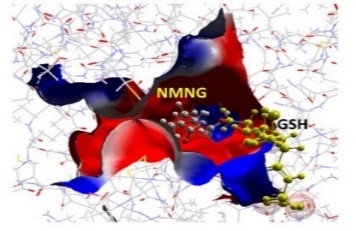  **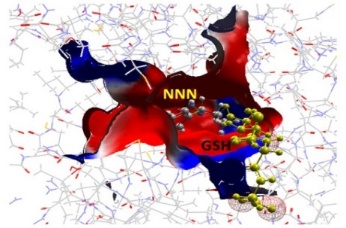**  **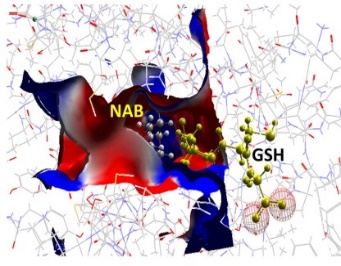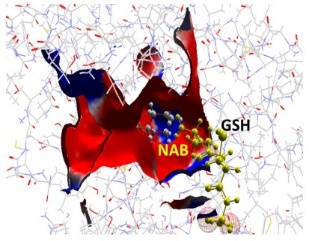** |
